# GP decisions to participate in emergencies: a randomised vignette study

**DOI:** 10.3399/bjgpopen20X101153

**Published:** 2021-01-20

**Authors:** Magnus Hjortdahl, Dorte Gyrd-Hansen, Peder A Halvorsen

**Affiliations:** 1 Department of Community Medicine, UiT The Arctic University of Norway Faculty of Health Sciences, Tromsø, Norway; 2 Department of Public Health, University of Southern Denmark, Odense, Denmark

**Keywords:** primary health care, general practice, prehospital care, emergencies

## Abstract

**Background:**

GPs use their judgement on whether to participate in emergencies; however, little is known about how GPs make their decisions on emergency participation.

**Aim:**

To test whether GPs' participation in emergencies is associated with cause of symptoms, distance to the patient, other patients waiting, and out-of-hours (OOH) clinic characteristics.

**Design & setting:**

An online survey was sent to all GPs in Norway (*n* = 4701).

**Method:**

GPs were randomised to vignettes describing a patient with acute shortness of breath and asked whether they would participate in a callout. The vignettes varied with respect to cause of symptoms (trauma versus illness), distance to the patient (15 minutes versus 45 minutes), and other patients waiting at the OOH clinic (crowding versus no crowding). The survey included questions about OOH clinic characteristics.

**Results:**

Of the 1013 GPs (22%) who responded, 76% reported that they would participate. The proportion was higher in trauma (83% versus 69%, χ^2^ 24.8, *P*<0.001), short distances (80% versus 71%, χ^2^ 9.5, *P*=0.002), and no crowding (81% versus 70% χ^2^ 14.6, *P*<0.001). Participation was associated with availability of a manned-response vehicle (adjusted odds ratio [OR] 2.06, 95% confidence interval [CI] = 1.25 to 3.41), and team training at the OOH clinic once a year (OR = 1.78, 95% CI = 1.12 to 2.82) or more than once a year (OR = 3.78, 95% CI = 1.64 to 8.68).

**Conclusion:**

GPs were less likely to participate in emergencies when the incident was not owing to trauma, was far away, and when other patients were waiting. A manned-response vehicle and regular team training were associated with increased participation.

## How this fits in

While GP participation in pre-hospital emergencies may contribute to better patient care, little is known about how GPs decide whether to participate or not. This study helps to address this knowledge gap. It examines how factors other than patient symptoms influence GPs’ decisions to participate in callouts using vignettes. It was found that GP participation was less likely when the incident was not owing to a trauma, was far away, and when other patients were waiting in the OOH clinic.

## Introduction

Norwegian regulations define an emergency as an acute onset or deterioration of disease or injury where prompt medical help can be decisive for the patient’s life and health.^1^ The national Emergency Medical Communication Centre (EMCC) is the first point of contact in life-threatening situations. The EMCC will then issue a 'red response' alert to the local OOH clinic and dispatch an ambulance to the scene. According to regulations, the GP on duty is expected to participate in such callouts whenever necessary. The judgement of whether it is necessary, however, is left to the GP’s discretion.^1^ The official policy in Norway is that GPs should participate on these callouts.^2^ However, GPs on call participate in about half of 'red response' callouts.^3,4^

The municipalities are legally bound to have at least one GP on call 24/7. Duty work at the local OOH clinic is a part of working as a regular GP in Norway.^1^ In some OOH clinics, the GPs get a fixed salary, in others they are payed according to a blended scheme with salary and fee for service. The municipality is responsible for equipment, staffing, and management at the OOH clinics. GPs will occasionally encounter emergencies during their normal office hours too, but then they are not connected to the EMCC and not expected to attend emergencies away from their office.

Studies have shown that GPs in Ireland, Canada, and Australia encounter a variety of emergencies and perform a wide range of interventions.^5–7^ In another study from Ireland, 36% of GP practices were involved in a cardiac arrest during a 5-year period. Both rural and urban GPs tended to cardiac arrests.^8^ In England and Sweden, researchers have found that GP involvement in emergencies can reduce hospital admissions, reduce costs, and improve quality of care.^9,10^

Little is known about how GPs decide whether to participate in callouts. A previous study found that GPs perceive such decisions as difficult, and that different GPs assess the same information in red alerts differently.^11^ Based on qualitative interviews with emergency medical technicians (EMTs) and GPs, it was hypothesised that factors not directly linked to the patient’s condition might influence this decision.^11,12^ An interview study of 47 GPs in the western part of Norway found that being occupied with other patients at the OOH clinic and distance to the patient were reasons for not participating on red response alerts.^13^

The primary aim of this study was to test — using a randomised design — whether distance to the patient, crowding at the OOH clinic, and cause of symptoms (trauma versus acute illness) might influence GPs’ decisions to participate in red alert callouts. A secondary aim was to examine whether participation in emergencies is associated with OOH clinic characteristics.

## Method

### Participants and data collection

In August 2016, all GPs in Norway registered by the Norwegian Health Economics Administration (HELFO) database (*n* = 4701) were invited to participate in an online survey. The invitation and two reminders were sent by mail. The Norwegian Centre of Rural Medicine used a dedicated Facebook group to encourage GP participation.

### Questionnaire

The GPs were presented with a vignette describing a red alert from the EMCC regarding a patient suffering from acute shortness of breath and were asked whether they would participate in an ambulance callout (Boxes 1 and 2). The vignettes varied with respect to cause of symptoms (trauma versus acute illness), the distance to the patient (15 versus 45 minutes), and patients waiting to be examined at the OOH clinic (crowding versus no crowding). This yielded 2 x 2 x 2 = 8 versions of the vignette (Table 1), and each participant was randomly allocated to one version only. The GPs were not informed about the randomisation. The questionnaire also included sociodemographic questions and organisational aspects regarding the GPs’ local OOH clinics. The questionnaire and vignettes were developed based on previous qualitative studies with GPs and EMTs.^11,12^

Box 1 **Example vignette (vignette group number seven). The information that differs between vignettes is put in boldface in this example.**

You are on duty at your local OOH clinic. It is a weekday and the time is 7pm. You are the only physician on call, **there are several paitents waiting to be examined**. The alarm sounds.

**'The patient is a 60-year-old man complaining of acute breathing difficulties**. He has rapid breathing and problems with speaking complete sentences. He is awake. The patient is located **45 minutes’ drive** from the OOH clinic.

Would you participate in this callout?

Box 2 **Example vignette (vignette group number one). The information that differs between vignettes is put in boldface in this example.**

You are on duty at your local OOH clinic. It is a weekday and the time is 7pm. You are the only physician on call, **it is quiet and you are doing paperwork**. The alarm sounds.

**There has been a car accident on an 80 kilometers per hour road. There is one injured person. The patient is a 60-year-old man complaining of acute breathing difficulties**. He has rapid breathing and problems with speaking complete sentences. He is awake.

The patient is located **15 minutes’ drive** from the OOH clinic.

Would you participate in this callout?

**Table 1.** Vignette groups

Vignette group	Combination of cause, distance, and crowding
1	Trauma condition, short distance, no crowding
2	Trauma condition, short distance, crowding
3	Trauma condition, long distance, no crowding
4	Trauma condition, long distance, crowding
5	Medical condition, short distance, no crowding
6	Medical condition, short distance, crowding
7	Medical condition, long distance, no crowding
8	Medical condition, long distance, crowding

### Outcome measure

The primary outcome measure was the proportion of GPs reporting that they would participate in the callout (that is, by attending the patient). Possible response options were 'yes' and 'no'.

### Independent variables

The main independent variables were cause of dyspnoea (trauma versus medical condition), distance to patient (15 minutes versus 45 minutes), and crowding at the OOH clinic (other patients waiting versus no patients waiting). Independent variables of secondary interest were if the OOH clinic was co-located with the ambulance service (yes or no), if the OOH clinic had a dedicated response vehicle (dedicated vehicle with driver, dedicated vehicle without driver, or no dedicated vehicle), distance to nearest hospital (more than 60 minutes’ drive, or less than 60 minutes’ drive), and if the OOH clinic had team training with the ambulance service (never or not relevant, less than annual, annual, several times a year).

### Statistical analysis

GP characteristics were described using means and percentages. The hypothesis was tested that the proportion of GPs attending to the hypothetical callout differed between the eight versions of the vignette using Pearson’s χ^2^ test. Multivariable logistic regression was used to explore possible associations between the participation in callout and OOH clinic characteristics.

Analyses were performed using SPSS statistics (version 26). *P* values <0.05 were considered statistically significant. Authors MH and PH analysed the data independently.

## Results

Of the 4701 GPs invited, 1013 responded, giving a response rate of 22%. Eleven responders were excluded from further analyses because they were no longer GPs, leaving a total of 1002 responders. The sample was fairly representative of Norwegian GPs with respect to age, sex, number of patients on the GP’s list, and specialist status (Table 2). Twenty-six per cent of the responders worked at an OOH clinic more than a 1 hour’s drive from the nearest hospital. Since 19% of Norwegian GPs work in rural municipalities,^14^ rural doctors were slightly over-represented. The eight randomised groups were fairly balanced with respect to age, sex, length of patient list, specialist status, and distance to nearest hospital.

**Table 2.** Characteristics of responders in the eight vignette groups

Vignette group	Mean age, years	Female, *n* (%)	Mean number ofpatients on GP list	Specialist in generalpractice, *n* (%)	Rural (>1 hour’s drive to nearest hospital), *n* (%)
1 (*n* = 113)	44	49/112 (44)	1024	66/113 (58)	24/108 (22)
2 (*n* = 136)	45	70/135 (52)	1034	71/136 (52)	39/131 (30)
3 (*n* = 125)	45	61/125 (49)	1070	78/125 (62)	28/119 (24)
4 (*n* = 109)	44	42/106 (40)	1011	62/107 (58)	23/103 (22)
5 (*n* = 127)	45	60/125 (48)	1038	79/125 (63)	28/122 (23)
6 (*n* = 118)	45	53/116 (46)	1038	67/116 (58)	35/114 (31)
7 (*n* = 132)	45	44/129 (34)	1073	72/130 (55)	31/127 (24)
8 (*n* = 142)	45	57/140 (41)	1053	73/140 (52)	39/141 (28)
Responders*n* = 1002	45	436/988 (44)	1043	568/992 (57)	247/965 (26)
All GPs in Norway in 2016(*n* = 4644)^a^	48	42%	1128	55%	19%

a Statistics Norway (SSB).

The majority (76%) of the participants reported that they would participate in the callout. Across the eight scenarios, the proportion that would participate in the callout varied from 59% to 87% (Table 3).

**Table 3.** Vignettes ranked in order of participation rates (from high to low)

Vignette group(combination of condition, distance, and crowding)	GPs participating on callout in the vignette, *n* (%)	Missing, *n*
Trauma, short distance, crowding	115/132 (87%)	4
Trauma, short distance, no crowding	98/113 (87%)	0
Medical condition, short distance, no crowding	105/123 (85%)	4
Trauma, long distance, no crowding	101/125 (81%)	0
Trauma, long distance, crowding	80/107 (75%)	2
Medical condition, long distance, no crowding	92/129 (71%)	3
Medical condition, long distance, crowding	84/139 (60%)	3
Medical condition, short distance, crowding	70/118 (59%)	0
Total	745/986 (76%)	16

Difference between scenarios: χ^2^ 60.8, degrees of freedom (DF) 7, *P*<0.05.

Participation in the callout was more likely when the patient had sustained a trauma, distance to the patient was short, and there was no crowding at the OOH clinic (Table 4).

**Table 4.** Participation rate related to condition, distance, and crowding

Variables	Proportion of GPs participating on the callout in the vignette, *n* (%)	χ^2^ value/DF	*P* value
Trauma condition	394/477 (83%)	24.8/1	<0.001
Medical condition	351/509 (69%)		
Short distance	388/486 (80%)	9.5/1	0.002
Long distance	357/500 (71%)		
No crowding	396/490 (81%)	14.6/1	<0.001
Crowding	349/496 (70%)		
All	745/986 (76%)		

DF = degrees of freedom.

Participation in the callout was associated with working at an OOH clinic that was equipped with a response vehicle manned by a dedicated driver (adjusted OR = 2.06, 95% CI = 1.25 to 3.41), and working at an OOH clinic that conducts team training once a year (OR = 1.78, 95% CI = 1.12 to 2.82) or more often than once a year (OR = 3.78, 95% CI = 1.64 to 8.68) compared with less than once a year (Table 5).

**Table 5.** Multivariable logistic regression analyses: associations between GPs participating on callout in vignettes and OOH clinic characteristics

	GPs participating on callout in vignette, *n* (%)	OR crude (95 % CI)	OR adjusted (95% CI)
**C** **o-located ambulance**	694/925 (75%)		
YesNo	277/357 (78%)417/568 (73%)	0.80 (0.59 to 1.09)Ref	0.88 (0.63 to 1.23) Ref
**Dedicated response vehicle**	703/937 (75%)		
Yes, with driverYes, without driverNo	138/167 (83%)123/163 (76%)442/607 (73%)	1.78 (1.15 to 2.75)^a^1.15 (0.77 to 1.71)Ref	2.06 (1.25 to 3.41)^a^ 0.92 (0.60 to 1.42) Ref
**Distance to hospital**	715/953 (75%)		
More than 60 minutesLess than 60 minutes	200/244 (82%)515/709 (73%)	1.71 (1.19 to 2.47)^a^Ref	1.47 (0.97 to 2.23) Ref
**Team training**	730/968 (75%)		
Never or not relevantLess than annuallyAnnuallySeveral times a year	300/436 (69%)196/257 (76%)167/200 (84%)67/75 (89%)	Ref1.46 (1.03 to 2.07)^a^2.29 (1.50 to 3.51)^a^3.80 (1.77 to 8.12)^a^	Ref 1.44 (0.98 to 2.11) 1.78 (1.12 to 2.82)^a^ 3.78 (1.64 to 8.68)^a^

Also adjusted for sex, age, specialist status, and OOH clinic location (large city: Oslo, Bergen, Stavanger, or Trondheim; single municipality or inter municipality).

a *P*<0.05.

## Discussion

### Summary

The majority of the responders would participate in the hypothetical callout to a patient with severe breathing difficulties. Participation in the callout was more likely when the patient had sustained a trauma, distance to the patient was short, and there was no crowding at the OOH clinic. GP participation was associated with working at an OOH clinic equipped with a manned-response vehicle and regular team training.

### Strengths and limitations

The main strengths of the study are the fairly large sample and the randomised design. However, there are important limitations. First, the findings are based on vignettes. Whether vignette-based studies can be considered representative of real-life practice is a long-standing concern. However, there is evidence that they compare favourably to studies based on standardised patient techniques, claims data, and medical record abstraction.^15–18^ In any case, it would hardly be possible to design a similar randomised controlled trial in a real-life setting. Twenty-two per cent of the GPs participated in the survey. A higher response rate would have been desirable, but relatively low response rates among GPs are often observed.^19^ The sample was fairly representative of the GP population with respect to age, sex, specialty attainment, and list size. A slightly higher number of rural GPs in the sample should be taken into consideration when interpreting the findings. Further, the possibility that GPs are unrepresentative with respect to unobservable characteristics cannot be excluded.

### Comparison with existing literature

Seventy-six per cent of the GPs reported that they would participate in the callout, which is high compared with studies and reports indicating that Norwegian GPs participate in 50% to 60% of callouts.^3,4,13^ The reason for the difference between this vignette study and observational studies may be that in real life the GPs receive alerts about a multitude of conditions, including several where they do not see the need for GP participation.^11^ In contrast, the clinical setting in the vignette was perceived as relevant by most GPs. Rural GPs were slightly over-represented in this study, and it may be speculated that they are more engaged in pre-hospital emergency medicine than the average GP. Another reason might be that the GPs in the present study over-reported intention to participate in the callout, perhaps motivated by social desirability.^20^ In a review, 43% of health-related studies using questionnaires were found to be influenced by social desirability.^21^

The present study suggests that distance to the emergency incident may affect GP participation in callouts. The findings corroborate with a previous Norwegian study where utilisation of OOH services was inversely associated with distance from the OOH clinic. This association was also found in patients with severe symptoms.^22^

The present study found that patients waiting to be examined at the OOH clinic lead to less GP involvement on callouts, suggesting that GPs are aware of the opportunity costs in terms of reduced health services provided to other patients. In a previous qualitative study, some of the GPs argued they often would be needed more at the OOH clinic, and that the EMTs are often competent to handle the callout themselves.^11^ The negative effect of crowding on patient treatment and outcome in emergency departments is well established.^23^

More GPs participated on the callout when the patient had breathing difficulties related to trauma. This is aligned with the results of the authors' previous qualitative study, where GPs reported greater inclination to participate when the alert suggested a dramatic situation such as a trauma.^11^ Breathing difficulties in trauma patients is a potential time critical, life-threatening symptom. One could argue that these patients would be more likely to benefit from GP participation. However, a recent study has shown that non-traumatic breathing difficulties may be at least as severe as trauma, and that non-traumatic breathing problems may pose greater diagnostic and therapeutic challenges in the pre-hospital setting.^24,25^ Consequently, the non-traumatic patients might benefit more from the GP's medical knowledge. According to decisionmaking theory, intuitive decisions are made based on experience and pattern recognition or analytical decisions by analysing the information thoroughly.^26^ As the decision whether to participate is time critical and based on limited information, it will often be an intuitive decision. However, improving decisions in this environment is not a trivial task.

Callout participation was also associated with working at an OOH clinic equipped with a manned-response vehicle. Fifty per cent of OOH clinics in Norway claim to have a dedicated response vehicle, but it is not known how many of these have drivers.^27^ Experiences from several OOH clinics in Norway suggest that introducing a GP-manned response vehicle with a dedicated driver leads to fewer hospital admissions.^2^

Team training had the strongest association with GP callout participation. The direction of this association is not obvious; it could go both ways. Pre-hospital emergency team training is mandatory in Norway. There are several benefits of simulation-based team training, including better patient treatment,^28^ as well as learning and leadership practices.^29,30^ Furthermore, pre-hospital personnel want to participate in team training.^11,12^ Despite this, only about 60% of Norwegian OOH clinics report annual training activities, and it is not known how often the individual healthcare worker participates.^4^

### Implications for research and practice

The results raise several questions when designing a pre-hospital emergency medicine system with strong GP involvement.

First, should GPs be allowed to decide whether to participate on callouts, or should this decision be made by the EMCC? In a qualitative study from Norway, GPs reported that they wanted to keep this authority as they perceived the GP to be best suited for making the decision in the given context.^11^ However, GP skills may not be needed in all emergencies. In a qualitative study, Norwegian EMTs suggested that GP contribution is most important in patients with non-specific symptoms, geriatric patients, children, and psychiatric conditions.^12^ Accurate dispatch from physician-staffed EMS was one of the top five research priorities in physician-provided pre-hospital critical care, according to a European research collaboration.^31^ There is, however, limited knowledge about which criteria accurately identify patients requiring advanced care in pre-hospital medicine.^32^

Second, since distance to the incident and OOH clinic crowding were associated with less GP involvement in hypothetical callouts, to what extent should OOH clinics be centralised? Western European countries have traditionally had a wide variety of organisation of OOH services.^33^ In countries such as the UK, Netherlands, and Denmark, there has been a shift towards centralisation of OOH clinics and increased use of triage and advice by telephone. If patients waiting to be examined at the OOH clinic affects the GPs’ decision to participate in callouts, OOH clinics may need to be better staffed. Alternatively, measures might be taken to avoid unnecessary contacts to the OOH clinic, such as having patients call ahead for advice, and triage by phone as in the Netherlands and Denmark, or at arrival at the OOH clinic.^34^

The findings should be investigated further, as they are based on hypothetical vignettes in questionnaires. Real-life data from the EMCCs may be used to study associations between GP participation and distance, type of emergency, and availability of response vehicles, respectively. It would also be interesting to design a project implementing team training in areas that do not train and measure if this affects GP participation on callouts. Ultimately, studies of associations between GP participation and patient outcomes would be desirable.

In conclusion, GPs were less likely to participate in hypothetical emergency callouts when the incident was not owing to a trauma, was far away, and when other patients were waiting in the OOH clinic. The availability of a response vehicle with a driver and regular team training were associated with increased participation in the callouts.
